# A Unique Arc of Bühler Variant Connecting the Arteries of the Foregut, Midgut, and Hindgut and Its Surgical Significance

**DOI:** 10.7759/cureus.42611

**Published:** 2023-07-28

**Authors:** Kamal A Abouzaid, Axel B Lichtenberg, Ahmad Y Karim, Emily R Stack, Mohammad Algoul, Ahmad Imam, Alexandra A Macpherson, Christina V Chanthanivong

**Affiliations:** 1 Department of Anatomical Sciences, William Carey University College of Osteopathic Medicine, Hattiesburg, USA; 2 Department of Emergency Medicine, University of Mississippi Medical Center, Jackson, USA; 3 Department of Internal Medicine, Baylor Scott & White All Saints Medical Center, Fort Worth, USA

**Keywords:** tandler longitudinal anastomosis, anastomotic channels between the vessels of the gut, vascular variation, celiomesenteric and intermesenteric vascular arcades, arc of buhler, vascular anomaly, vascular imaging, cadaver case report, surgical vascular complications, clinical and functional anatomy

## Abstract

This case report presents a rare variation of the arc of Bühler (AOB) in a cadaver during the abdominal dissection assignment in the Ross Anatomy Lab at William Carey University College of Osteopathic Medicine. The AOB is a patent anastomotic channel between the celiac trunk and the superior mesenteric artery independent of the gastroduodenal artery and dorsal pancreatic artery. This report describes in detail a complex and extensive branching pattern of a unique AOB variant. Our findings contribute to the limited literature on this condition and emphasize the importance of thorough knowledge of vascular variations to avoid potential complications during surgical procedures.

## Introduction

The arc of Bühler (AOB), according to Tandler, is an anastomosis between the celiac trunk (CT) and the superior mesenteric artery (SMA) [1]. Tandler's longitudinal anastomosis theory is typically used to explain the various anatomical variations observed in the splanchnic branches of the abdominal aorta [2]. The embryological origin of the splanchnic blood vessels is a result of intricate developmental changes in 10-22 ventral segmental arteries. As the embryo matures, the roots of the 11th and 12th ventral segmental arteries regress and gradually disappear. However, the 10th ventral segmental artery persists as the CT, and the SMA develops from the persistent 13th segmental artery. During this developmental process, a ventral longitudinal anastomosis may remain intact, serving as a direct anastomosis between the celiac artery and the SMA. This persistent anastomosis forms the AOB [3]. Based on a recent systematic review, the AOB is considered an uncommon vascular anomaly with a prevalence that ranges between 1% and 4% with an average of 1.7% [4]. 

Expanding upon the existing understanding of the AOB as a rare developmental anomaly, this case report provides novel insights into an exceptional branching pattern of the AOB that supplies the foregut, midgut, and hindgut. By documenting this unique variation of the arc, this report contributes to the limited body of scientific literature, emphasizing the importance of an in-depth comprehension of vascular variations in order to optimize surgical outcomes and mitigate potential complications.

## Case presentation

**Figure 1:**
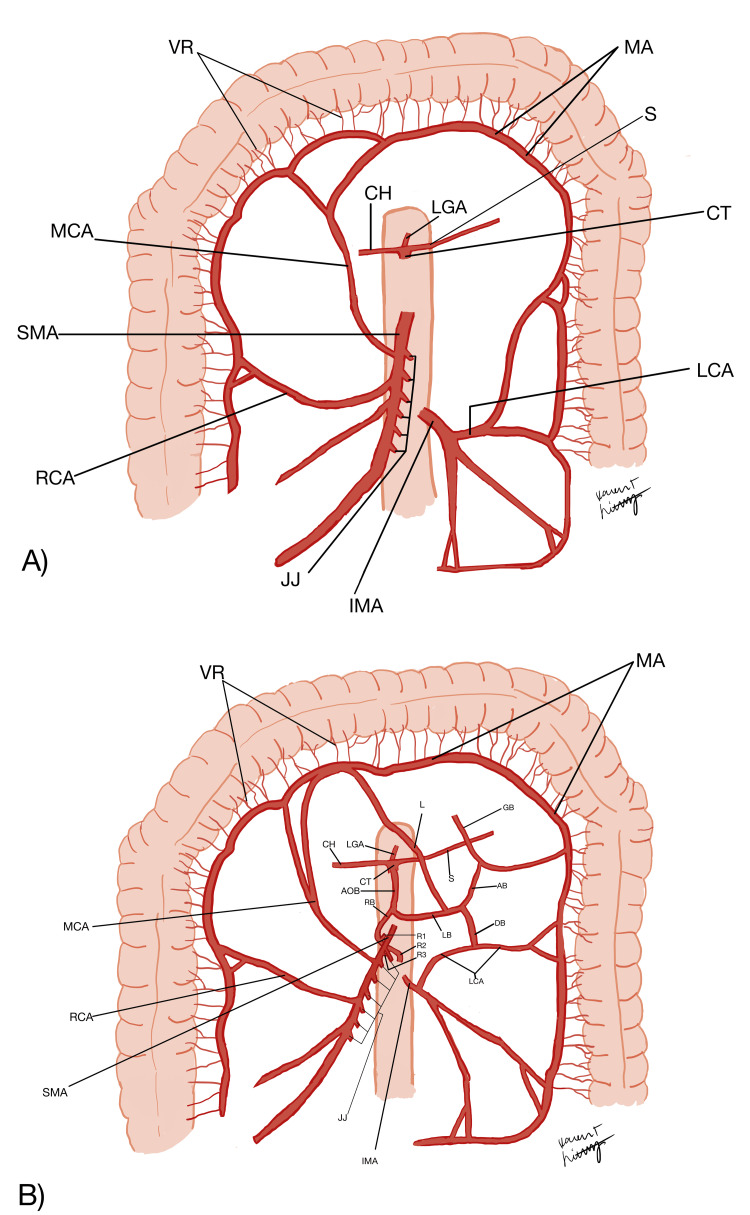
A schematic of the normal mesenteric arteries for comparison (A), and the AOB variation seen in this case (B). Image credits: Karen Lichtenberg AB: Ascending branch of AOB; AOB: arc of Bühler; CH: common hepatic artery; CT: celiac trunk; DB: descending branch of AOB; IMA: inferior mesenteric artery; JJ: jejunal branches of SMA; L: left branch of AOB anastomosing with the MCA; LB: left branch of AOB; MCA: middle colic artery; LCA: left colic artery; LGA: left gastric artery; MA: marginal artery; RB: right branch of AOB; R1: pancreatic branch; R2: pancreatic and jejunal branch; R3: anastomosis branch with the SMA which appears as a continuation of the AOB; S: splenic artery; SMA: superior mesenteric artery; VR: vasa recta.

**Figure 2:**
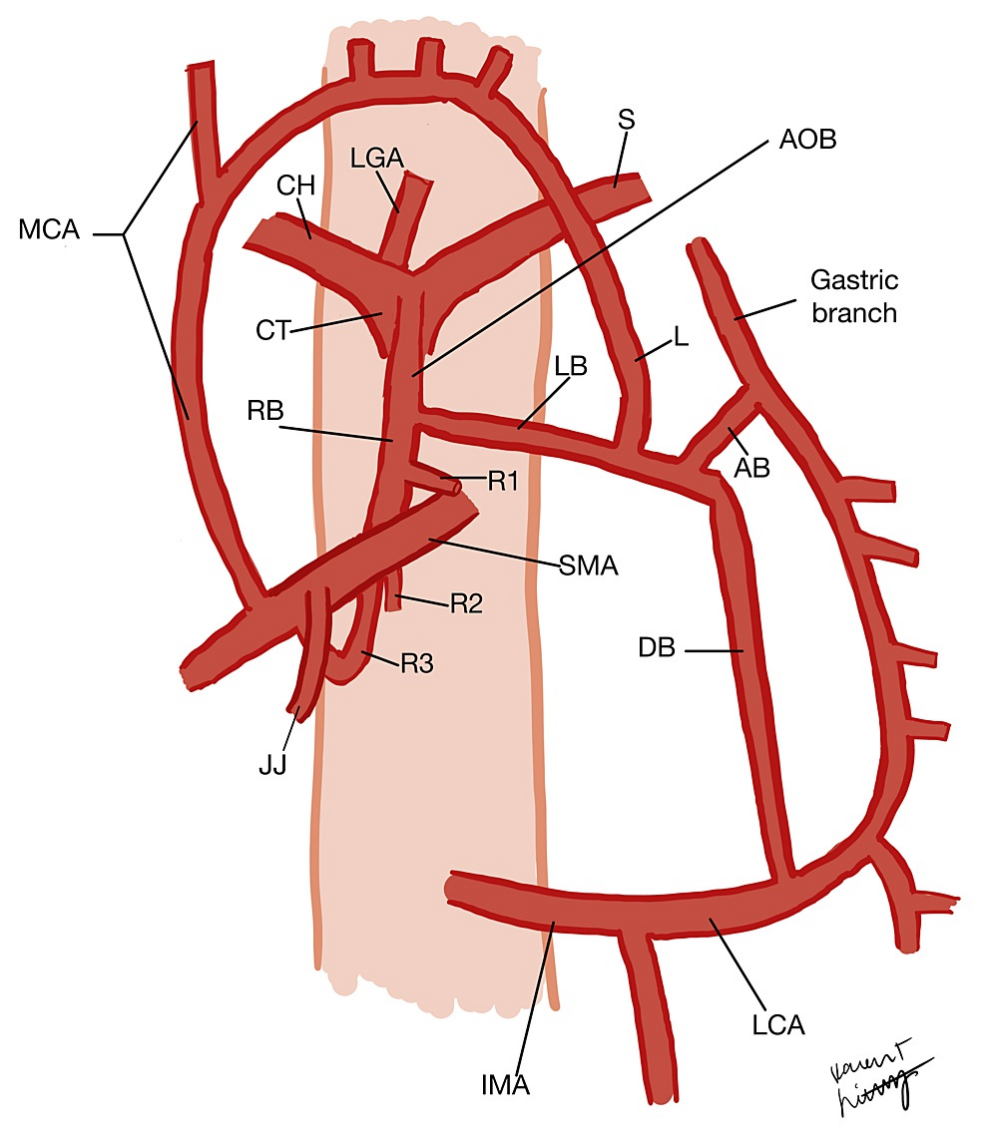
A schematic showing a close-up view of the AOB variation seen in this case. Note that the size of the arteries has been adjusted to facilitate easier visualization of their connections in relation to each other. Image credits: Karen Lichtenberg AB: Ascending branch of AOB; AOB: arc of Bühler; CH: common hepatic artery; CT: celiac trunk; DB: descending branch of AOB; IMA: inferior mesenteric artery; JJ: jejunal branches of SMA; L: left branch of AOB anastomosing with the MCA; LB: left branch of AOB; LCA: left colic artery; LGA: left gastric artery; MCA: middle colic artery; RB: right branch of AOB; R1: pancreatic branch; R2: pancreatic and jejunal branch; R3: anastomosis branch with the SMA which appears as a continuation of the AOB; S: splenic artery; SMA: superior mesenteric artery.

The AOB variant was observed, dissected, and examined in a 76-year-old Caucasian male cadaver during the dissection laboratory at William Carey University College of Osteopathic Medicine. This congenital anomaly was identified in one out of 24 cadavers (4.2%). The cadaveric donors were received from the University of Southern Alabama Anatomical Gift Program. Schematic images have been produced to depict and delineate the unique branching patterns of the AOB in this cadaveric donor (Figures 1, 2).

**Figure 3:**
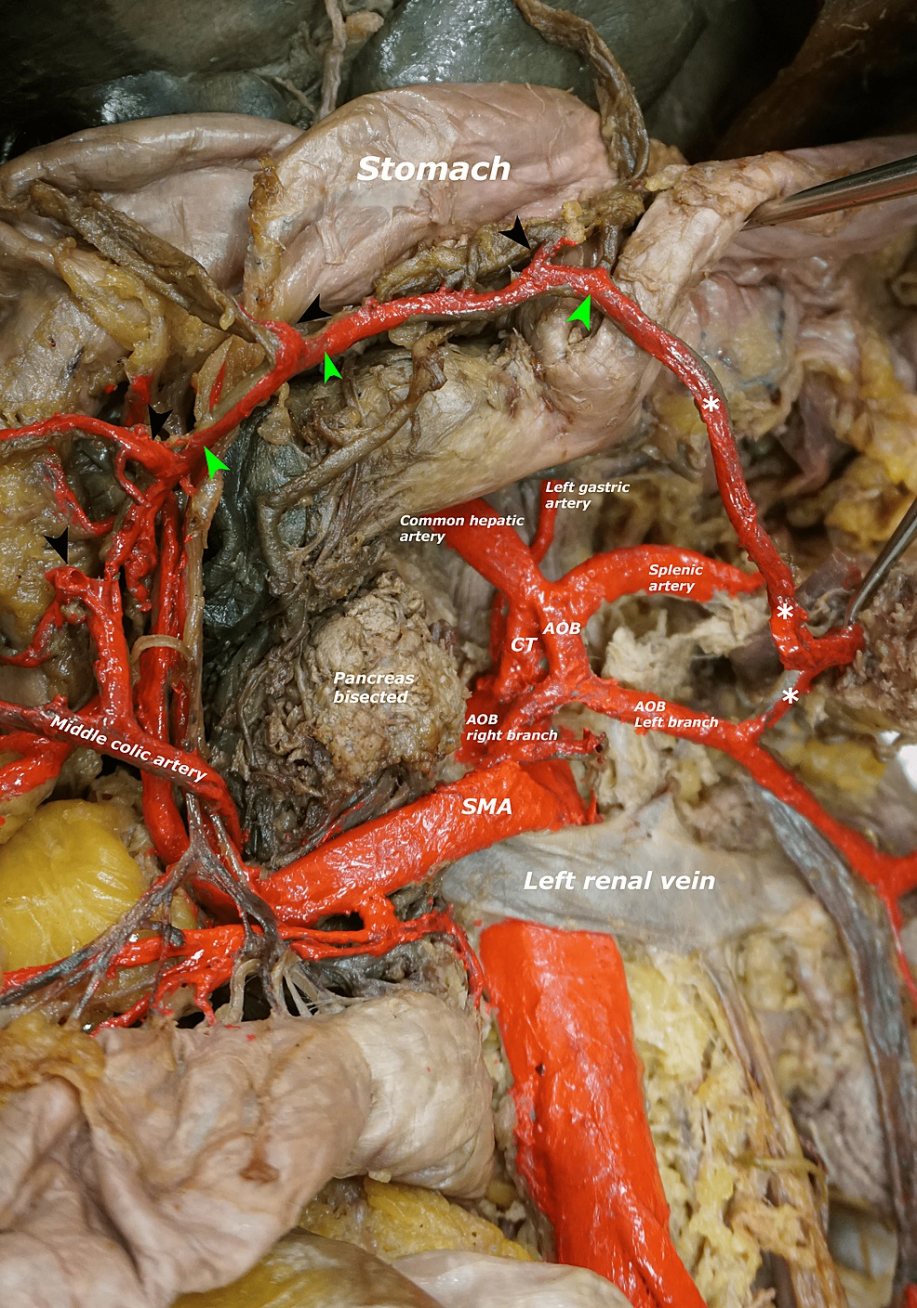
The branching pattern of AOB. Black arrowheads: vasa recta (cut); green arrowheads: marginal artery; asterisks: left branch of AOB anastomosing with the MCA; AOB: arc of Bühler; CT: celiac trunk; SMA: superior mesenteric artery; MCA: middle colic artery.

To expose the pancreas via the lesser sac, the greater omentum was removed, and the transverse colon was ligated and transected at the hepatic and splenic flexures and removed. The neck of the pancreas was then bisected and reflected to visualize the course and branching of the AOB deep to the pancreas (Figure 3). The CT, the SMA, the AOB, and their branches were then stained with red nail polish for better visualization. To remove the block of abdominal viscera and their blood supply, we began by tying and transecting the descending colon at the sigmoid colon. The aorta was then transected just distal to the diaphragm and the common iliac arteries were transected distal to the origin. The inferior vena cava was also divided just inferior to the liver and at its formation. The stomach, duodenum, jejunum, ileum, descending colon, pancreas, spleen, and kidneys, along with their blood vessels, were then removed from the cadaver. 

**Figure 4:**
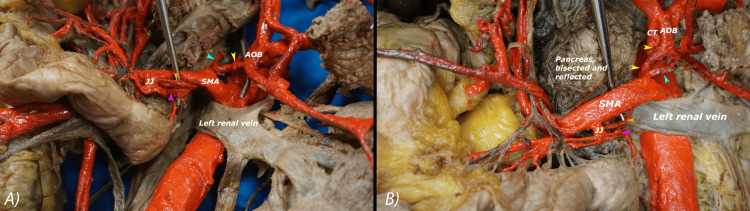
The three branches of the right branch of the AOB are shown in these two images. The three branches are shown in relation to the SMA (A). A probe has been used to move the SMA to reveal better visualization of the three branches of the right branch of AOB (B). Yellow arrowheads: Right branch of AOB; blue arrowhead: pancreatic branch; pink arrowhead: branch to head and uncinate process of the pancreas and jejunum; orange arrowhead: branch that anastomoses with the SMA; AOB: arc of Bühler; CT: celiac trunk; JJ: jejunal branches of SMA; SMA: superior mesenteric artery.

The AOB originates from the inferior aspect of the CT and descends inferiorly for 1.2 cm before bifurcating into a right and left branch. The right branch courses inferiorly and to the right for 1.7 cm and divides into three branches deep to the SMA (Figure 4). One branch runs toward the pancreas and had been severed during the bisection of the pancreas. It appears to contribute to the anastomosis around the pancreas. The second branch runs inferiorly for 3.9 cm and divides into several branches that supply the head and uncinate process of the pancreas and the jejunum. The third branch, which appears as a continuation of the AOB, runs inferiorly for 3.6 cm to connect with the SMA. The distance between the origin of the AOB from CT to its termination at the SMA is 7.2 cm.

**Figure 5:**
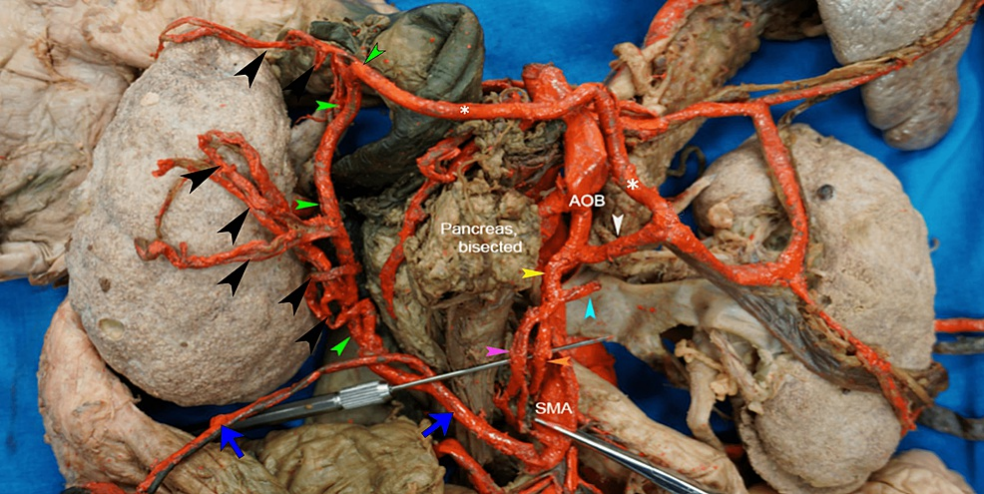
The anastomosis between the left branch of AOB and the middle colic artery. Black arrowheads: Vasa recta (cut); white arrowhead: left branch of AOB; asterisks: left branch of AOB anastomosing with middle colic artery; green arrowheads: marginal artery; yellow arrowhead: right branch of the AOB; blue arrowhead: pancreatic branch of right branch of AOB; pink arrowhead: pancreatic and jejunal branch of right branch of AOB; orange arrowhead: anastomotic branch with the SMA; dark blue arrows: middle colic artery; AOB: arc of Bühler; SMA: superior mesenteric artery.

**Figure 6:**
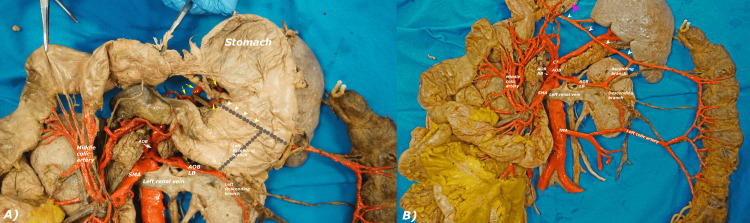
The branching pattern of the ascending branch of the left branch of the AOB is shown in these two images. The ascending branch of the left branch of the AOB is shown in reference to the stomach in anatomical position (A). The stomach is rotated 180 degrees to the right to show the ascending branch of the left branch of the AOB (B). In both images, the left branch of the AOB anastomosing with the middle colic artery has been displaced deep to the bisected pancreas for better visualization of this anastomotic channel. Dotted line:  Ascending branch of left branch of AOB that runs posterior to the stomach; white arrowheads: anastomotic channel connecting the ascending branch to the gastric arteries; yellow arrows: gastric arteries; pink arrowhead: lesser curvature of the stomach; AOB: arc of Bühler; AOB LB: arc of Bühler left branch; CT: celiac trunk; IMA: inferior mesenteric artery; SMA: superior mesenteric artery.

The left branch of the AOB courses inferiorly and to the left for 2.4 cm from the arc’s bifurcation. At this point, the left branch of the AOB gives off a branch that anastomoses with the middle colic artery (MCA) (Figure 5). Then the left branch of the AOB continues for 4.1 cm, descending deep to the transverse colon and in the retroperitoneal space of the left infra-colic compartment and divides into ascending and descending branches. The ascending branch supplies the proximal segment of the descending colon. Moreover, there is another anastomotic channel connecting the ascending branch to the gastric arteries at the lesser curvature of the stomach (Figure 6). The descending branch courses downward and to the left and anastomoses with the left colic artery (LCA).

## Discussion

In this case, we discovered a unique anastomotic channel that not only connects the CT and SMA, as typically seen with the AOB [1], but also bridges the arterial supply of the foregut, midgut, and hindgut by extending to the MCA, LCA, and gastric arteries. Although vascular variations are common, to our knowledge, this AOB variant has not previously been reported [5,6]. Douard et al. categorized variations of vascular anastomotic arcades according to Tandler's theory [2]. These include coeliomesenteric anastomosis, which can persist as the AOB connecting the CT and SMA, and intermesenteric anastomoses. Intermesenteric anastomoses comprise the Riolan arcade, connecting the right colic artery (from the SMA) and the LCA (from the inferior mesenteric artery (IMA)), and the Villemin arcade, potentially linking the IMA with the SMA [2]. 

One case report presented an AOB that bifurcates posterior to the pancreas and connects with the arc of Riolan [7]. The AOB has also been previously found to anastomose with the IMA, supplying circulation to the hindgut [8]. Another report describes the incidence of anastomosis connecting the foregut, midgut, and hindgut via the common hepatic artery and the dorsal pancreatic artery to the MCA, LCA, and IMA [9]. While the AOB in our case is similar to some other reports in which it supplies segments of the gut in addition to connecting the CT and SMA [7-9], our account of the AOB is unique in that it supplies the foregut, midgut, and hindgut through the MCA, LCA, and the gastric arteries. Moreover, the anastomotic branches observed in this case report demonstrate a higher complexity that underscores the remarkable adaptability of embryonic longitudinal anastomoses. This finding further emphasizes the significance of identifying arterial variations during preoperative diagnostic evaluation.

Multiple reports concerning celiac stenosis have emphasized the importance of maintaining the AOB for successful patient treatment and care [10,11]. For instance, Ochoa et al. described a case where a pancreaticoduodenectomy procedure resulted in celiac stenosis in a patient with median arcuate ligament syndrome. In this case, the high-grade stenosis was asymptomatic due to collateral circulation from the AOB to the celiac trunk distal to the stenosis [10]. Moreover, during liver graft procurement, the importance of being aware of the AOB to prevent potential ischemic complications and transplant rejection has been highlighted. In one case, a donor considered for a liver graft had a partially occluded CT that was salvaged by a significant AOB. The AOB was completely dissected with the liver graft to provide adequate perfusion, leading to a successful transplant [12]. Our specific AOB variation, however, introduces a unique scenario. With the additional gastric branch anastomosing with the lesser gastric arteries, our variation could potentially provide a more extensive collateral circulation to the celiac trunk, thereby further reducing the risk of ischemia and improving the patient outcome postsurgery.

While the AOB has primarily been described as a source of collateral circulation, it has also been reported as a potential site for aneurysms and can complicate routine procedures [1,12]. One report described an incidental case of an asymptomatic AOB aneurysm during abdominal computed tomography angiography, which was treated with transcatheter coil embolization [1]. Expanding on the diverse clinical applications of the AOB, another report highlighted its use in the chemoembolization treatment of hepatocellular carcinoma. In this case, where proximal CT occlusion would typically complicate the treatment, the AOB allows access to the proper hepatic artery, facilitating catheterization and tumor treatment [13]. The more complex branching pattern that was observed in the present case provides more extensive anastomoses between the arteries of the foregut, midgut, and hindgut. With the advancement of imaging modalities and the increase of endovascular interventions, it could be argued that the branches of this unique AOB variant could potentially provide an alternative access for endovascular procedures that target the SMA and the IMA in case these major mesenteric vessels are stenosed or obstructed.

## Conclusions

Our findings concur with previous reports that describe the AOB as an anastomotic channel between the CT and SMA. However, a key distinguishing feature for this case is that the branching pattern of the AOB is more complex than what has previously been reported. This current variant, bridging the arterial supply of the foregut, midgut, and hindgut, underscores the importance of screening for arterial variations in preoperative evaluations. We believe that the AOB is more common than expected and has frequently been missed during cadaveric dissections. Our findings contribute to the limited literature on this variation, emphasizing the need for more cadaveric research to identify and describe the prevalence and the possible branches of the AOB.

## References

[1] Jeong SJ, Lim NY, Jang NK, Choi SJ, Kim JK, Jeong YY, Kang HK (2008). Transcatheter coil embolization of an Arc of Buhler aneurysm. Korean J Radiol.

[2] Douard R, Chevallier JM, Delmas V, Cugnenc PH (2006). Clinical interest of digestive arterial trunk anastomoses. Surg Radiol Anat.

[3] McNulty JG, Hickey N, Khosa F, O'Brien P, O'Callaghan JP (2001). Surgical and radiological significance of variants of Bühler's anastomotic artery: a report of three cases. Surg Radiol Anat.

[4] Kowalczyk KA, Pękala J, Kawzowicz M, Pękala PA, Tomaszewski KA (2023). The meta-analysis and systematic review of prevalence and clinical anatomy of the arc of Buhler. Sci Rep.

[5] Lichtenberg A, Kim SJ, Rogers L (2023). Aberrant left colic artery and accessory right colic artery: a case report and surgical implications. Cureus.

[6] Sahani D, Saini S, Pena C (2002). Using multidetector CT for preoperative vascular evaluation of liver neoplasms: technique and results. AJR Am J Roentgenol.

[7] Pratap H, Ramamurthy S (2023). Bifurcated arc of Buhler coexistent with an arc of Riolan-a rare case report. J Clin Diagn Res.

[8] Gupta M, Agarwal S (2013). A direct anastomosis between the stems of celiac trunk and left colic artery by an anomalous fourth celiac trunk branch: first cadaveric study. Clin Anat.

[9] Janardhanan J, Nayak S, D’Costa S, Latha P, Mangala P, Rai R (2008). An anomalous digestive arterial anastomosis connecting foregut, midgut and hindgut. Trakya Universitesi Tip Fakultesi Dergisi.

[10] Ochoa JE, Pointer DT Jr, Hamner JB (2016). Vascular anomalies in pancreaticoduodenectomy: a lesson learned. Case Rep Surg.

[11] Baz RO, Scheau C, Baz RA, Niscoveanu C (2020). Buhler’s arc: an unexpected finding in a case of chronic abdominal pain. J Gastrointestin Liver Dis.

[12] Incarbone N, De Carlis R, Centonze L, Lauterio A, De Carlis L (2021). Discovery of a rare variant of the arc of Bühler during liver procurement. Exp Clin Transplant.

[13] Shah N, Chen O, Cohen GS (2010). Arc of Buhler catheterization for tumor therapy: case report. J Interv Oncol.

